# Complete Genome Sequence of Arcanobacterium phocisimile Strain DSM 26142, Isolated from a Harbor Seal

**DOI:** 10.1128/MRA.00215-21

**Published:** 2021-07-15

**Authors:** Maria Borowiak, Mazen Alssahen, Burkhard Malorny, Christoph Lämmler, Ursula Siebert, Madeleine Plötz, Amir Abdulmawjood

**Affiliations:** a German Federal Institute for Risk Assessment (BfR), Department for Biological Safety, Berlin, Germany; b Institut für Hygiene und Infektionskrankheiten der Tiere, Justus-Liebig-Universität Gießen, Gießen, Germany; c Institute for Terrestrial and Aquatic Wildlife Research (ITAW), University of Veterinary Medicine Hannover, Hannover, Germany; d Institute of Food Quality and Food Safety, University of Veterinary Medicine Hannover, Hannover, Germany; University of Delaware

## Abstract

Bacteria of the genus *Arcanobacterium* can be found in a variety of hosts. The species Arcanobacterium phocisimile was originally identified in a free-living harbor seal in the German North Sea in 2004. Here, we announce the complete genome sequence of Arcanobacterium phocisimile strain DSM 26142.

## ANNOUNCEMENT

The genus *Arcanobacterium* consists of facultative, anaerobic, asporogenous, Gram-positive bacteria (1). So far, 11 species have been described, namely, Arcanobacterium haemolyticum, A. phocae, A. pluranimalium, A. hippocoleae, A. canis, A. phocisimile, A. pinnipediorum, A. wilhelmae, A. urinimassiliense, A. ihumii, and A. bovis (according to NCBI Taxonomy at https://www.ncbi.nlm.nih.gov/Taxonomy/Browser/wwwtax.cgi?id=28263; 8 February 2021). In some cases, *Arcanobacterium* colonization could be directly linked to infectious diseases in humans and animals (2–4). For better insights into this potentially pathogenic genus, we aim to provide genome sequences for *Arcanobacterium* species for further characterization and phylogenetic classification. Here, we present a closed genome sequence for *A. phocisimile*.

Arcanobacterium phocisimile strain DSM 26142, originally published under the name 2698^T^, was isolated from a vaginal swab specimen from a free-living harbor seal of the German North Sea in 2004 during a monitoring program (1). The strain was cultivated for 48 h at 37°C under microaerobic conditions on sheep blood agar. Bacterial cells were scraped from the blood agar plate, and genomic DNA was extracted using the MagMAX Microbiome Ultra nucleic acid isolation kit (Thermo Fisher Scientific, Darmstadt, Germany). The genomic DNA was subjected to whole-genome sequencing using Illumina and Oxford Nanopore technologies.

An Illumina sequencing library was prepared using the Nextera DNA Flex kit (Illumina, San Diego, CA, USA). Sequencing was performed in 2 × 151 cycles on an Illumina NextSeq 500 sequencer using the NextSeq 500/550 midoutput kit v2.5. A MinIon sequencing library was prepared using the rapid barcoding kit (Oxford Nanopore Technologies [ONT], Oxford, UK) and sequenced on an ONT MinIon device connected to ONT MinIT (software v19.12.5, including Guppy basecaller v3.2.10) using a FLO-MIN106 R9 flow cell.

For bioinformatics analysis, software default parameters were applied unless otherwise noted. Illumina reads were trimmed using fastp v0.19.5 (5). After trimming, 2 million high-quality reads were available (Q30, ≥87.8%). Nanopore reads were trimmed and filtered using Porechop v0.2.3 (https://github.com/rrwick/Porechop) and NanoFilt v2.7.1 (6). Quality assessment using NanoStat v1.2.1 (6) revealed 43,918 reads with a read length *N*_50_ value of 9,162 bp and a mean read quality score of 10.9. Both data sets were *de novo* assembled and circularized with Unicycler v0.4.8, including Pilon (7–9). Subsequently, the genome sequence was annotated using Prokka v1.1.3 (https://github.com/tseemann/prokka). The annotated genome was imported into CLC Genomics Workbench v9.5.2. The annotations were searched for the *dnaA* gene, which was then used to manually define the starting point of the genome. The final genome sequence consists of a closed chromosome of 1,988,065 bp with a G+C content of 51.1%. The genome sequence was deposited in the NCBI Nucleotide Database and annotated using the Prokaryotic Genome Annotation Pipeline v4.11 (https://www.ncbi.nlm.nih.gov/genome/annotation_prok/) (10).

**FIG 1:**
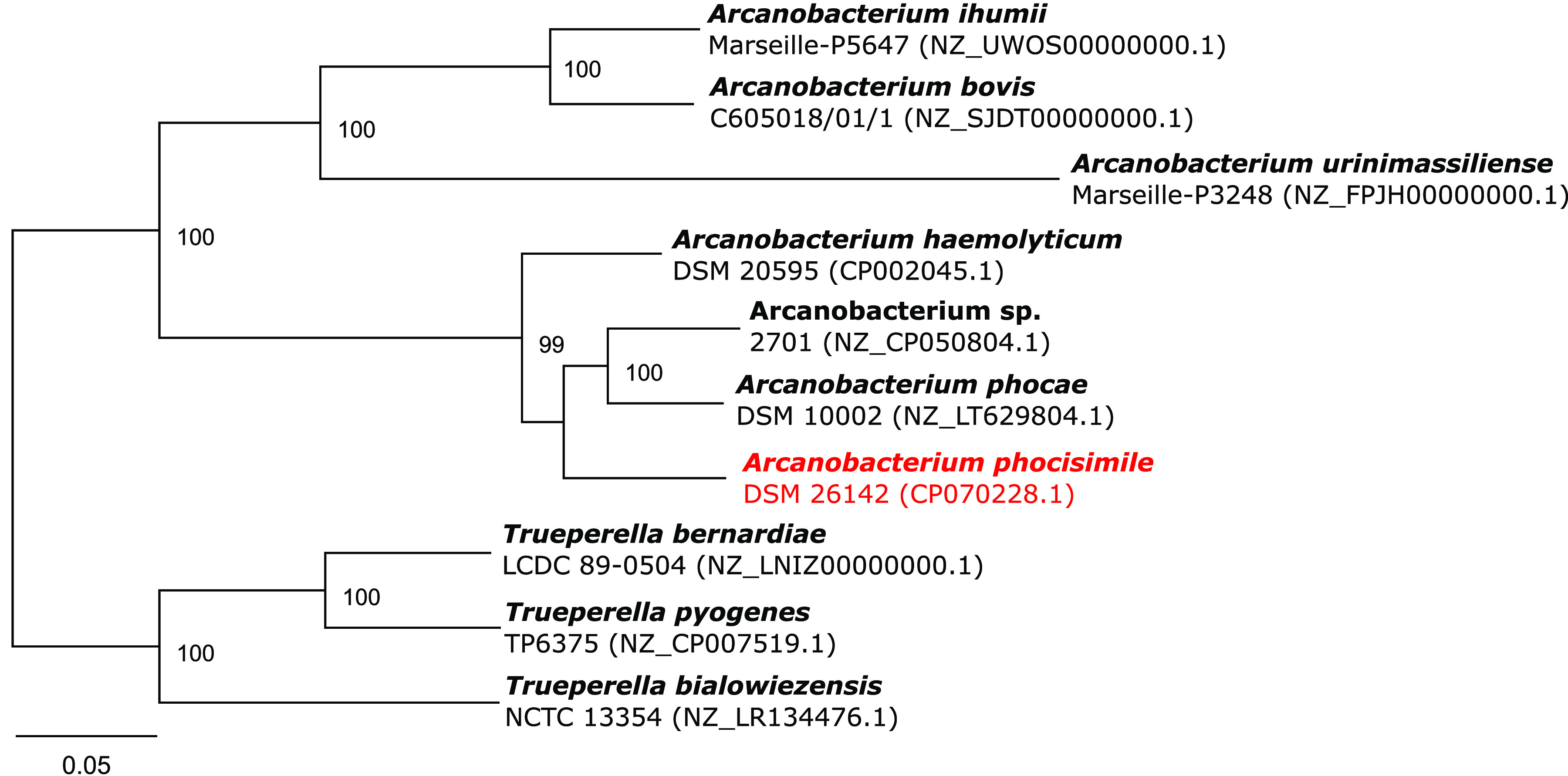
Best-scoring maximum likelihood tree based on the comparison of the amino acid sequences of 107 essential single-copy core genes of Arcanobacterium phocisimile strain DSM 26142, other published *Arcanobacterium* spp., and closely related *Trueperella* spp. with bcgTree. Numbers at branches designate bootstrap support values resulting from 100 bootstrap replicates.

Arcanobacterium phocisimile strain DSM 26142 was compared to previously published *Arcanobacterium* sp. and *Trueperella* sp. genomes locally annotated using Prokka v1.1.3. Phylogeny was inferred through amino acid sequence comparison of 107 single-copy core genes with bcgTree v1.1.0 (11). The resulting maximum likelihood tree including bootstrap values from 100 bootstrap repetitions was visualized in Geneious v2020.2.2 (Biomatters, Auckland, New Zealand), manually rooted using the *Trueperella* sp. node, and finalized using Inkscape v1.0.2. The final tree (Fig. 1) reveals that Arcanobacterium phocisimile strain DSM 26142 is most closely related to *A. phocae* DSM 10002 and *Arcanobacterium* sp. strain 2701.

### Data availability.

The complete genome sequence has been deposited in NCBI GenBank under the accession number CP070228.1. MinIon and Illumina sequencing data were deposited in the NCBI Sequence Read Archive (SRA) under the accession numbers SRX10068738 and SRX10068737, respectively.
